# Single‐cell RNA sequencing unveils an Odc1‐marked endothelial subpopulation critical for pathological angiogenesis

**DOI:** 10.1002/ctm2.1640

**Published:** 2024-03-27

**Authors:** Ming Tong, Yun Bai, Jiao Lyu, Ling Ren, Linyu Zhang, Mudi Yao, Xiaoyan Han, Xiumiao Li, Dan Li, Pei‐Quan Zhao, Qin Jiang, Biao Yan

**Affiliations:** ^1^ Eye Institute and Department of Ophthalmology Eye and ENT Hospital, State Key Laboratory of Medical Neurobiology Fudan University Shanghai China; ^2^ College of Information Science Shanghai Ocean University Shanghai China; ^3^ Department of Ophthalmology Xinhua Hospital Affiliated to Shanghai Jiaotong University School of Medicine Shanghai China; ^4^ The Fourth School of Clinical Medicine Nanjing Medical University Nanjing China; ^5^ The Affiliated Eye Hospital Nanjing Medical University Nanjing China; ^6^ Department of Ophthalmology Shanghai General Hospital, Shanghai Jiao Tong University School of Medicine Shanghai China

Dear Editor,

Understanding the molecular basis of endothelial heterogeneity can provide novel insights into an anti‐angiogenic treatment.^1^ Herein, we utilised single‐cell RNA sequencing to delineate endothelial heterogeneity in oxygen‐induced retinopathy mouse model (OIR), a model for retinal angiogenesis.^2^ Newborn C57BL/6J mouse pups underwent hyperoxia exposure (75% O_2_) from postnatal day 7 (P7) to P12. Next, they were transferred to room air for inducing retinal angiogenesis.^3^ scRNA‐seq analysis was conducted on retinas harvested from both non‐OIR mice and OIR mice at P17. Following scRNA‐seq data preprocessing, 42 772 high‐quality cell samples were obtained, comprising 22 534 cells from the non‐OIR group and 20 238 cells from the OIR group. Uniform Manifold Approximation and Projection (UMAP) algorithm was used for retinal cell annotation and different retinal markers were shown (Figure S1).

We next focused on endothelial cells (ECs) due to their critical roles in angiogenesis.^4^ We identified three different EC sub‐populations via UMAP reduction. The top 10 genes in each sub‐population were delineated (Figure 1A,B). Notably, EC sub‐population 2 was predominantly present in OIR retinas, prompting speculation about its involvement in angiogenesis (Figure 1C). The top 2 genes in different EC sub‐populations and canonical endothelial markers were listed (Figure 1D,E). Differentially expressed genes (DEGs) were identified between EC sub‐population 2 and other sub‐populations. Gene Ontology (GO), Kyoto Encyclopedia of Genes and Genomes (KEGG) and Reactome pathway analysis unveiled an association of EC sub‐population 2 with pathological angiogenesis. Additionally, we highlighted DEGs linked to ECM organisation (Col18a1, Col15a1, Pxdn and Loxl2)^5^ within EC sub‐population 2 (Figure S2).

**FIGURE 1:**
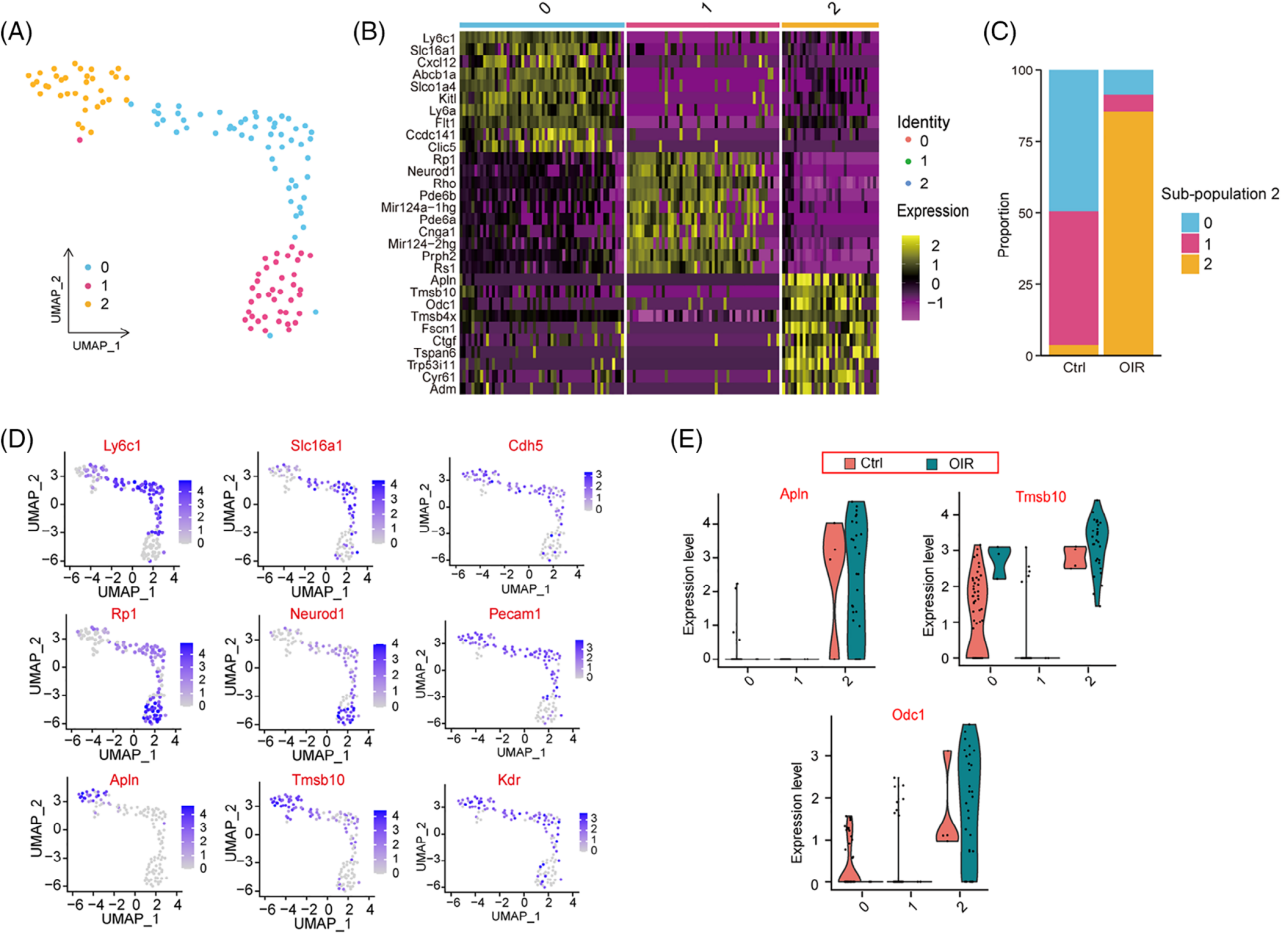
Transcriptomic heterogeneity of endothelial cells during pathological angiogenesis. (A) Endothelial cells (ECs) were colour‐coded by subpopulations and identified using the unsupervised graph‐based algorithm, UMAP. (B) The heatmap displayed the top 10 abundantly expressed genes in different EC sub‐populations. (C) A bar graph illustrated the percentage distribution of EC sub‐populations in OIR retinas and non‐OIR control retinas. (D) Dot plots presented the expression of the top two differentially expressed genes and canonical endothelial genes (Cdh5, Pecam1, and Kdr) in different EC sub‐populations. (E) Violin plots demonstrated the highest abundant gene in EC sub‐population 2 in both the OIR group and non‐OIR control group. UMAP, Uniform Manifold Approximation and Projection; OIR, oxygen‐induced retinopathy.

Odc1 gene aroused great interest due to its pronounced expression in EC sub‐population 2. The Odc1‐positive signal exhibited localisation at neovascular tufts within OIR retinas, co‐localising with vascular marker (IB4) and endothelial marker (CD31) (Figure S3). Notably, this signal did not exhibit co‐localisation with glutathione synthetase (GS), a glial marker, or NeuN, a marker for retinal ganglion cells (Figure S4).^6^, ^7^ Subsequently, we determined the role of Odc1 in endothelial angiogenic functions. Transfection of Odc1 siRNA significantly decreased Odc1 expression at mRNA and protein levels in ECs (Figure 2A,B). Odc1 silencing caused a marked reduction in endothelial proliferation, tube formation ability, migration ability and wound healing ability (Figure 2C–F), indicating that Odc1 silencing exerts an anti‐angiogenic effect. Additionally, we employed α‐difluoromethylornithine (DFMO) for pharmacological intervention of the Odc1 activity.^8^ DFMO administration inhibited endothelial proliferation, tube formation, migration, and wound healing ability, demonstrating a similar anti‐angiogenic effect as Odc1 silencing (Figure S5).

**FIGURE 2:**
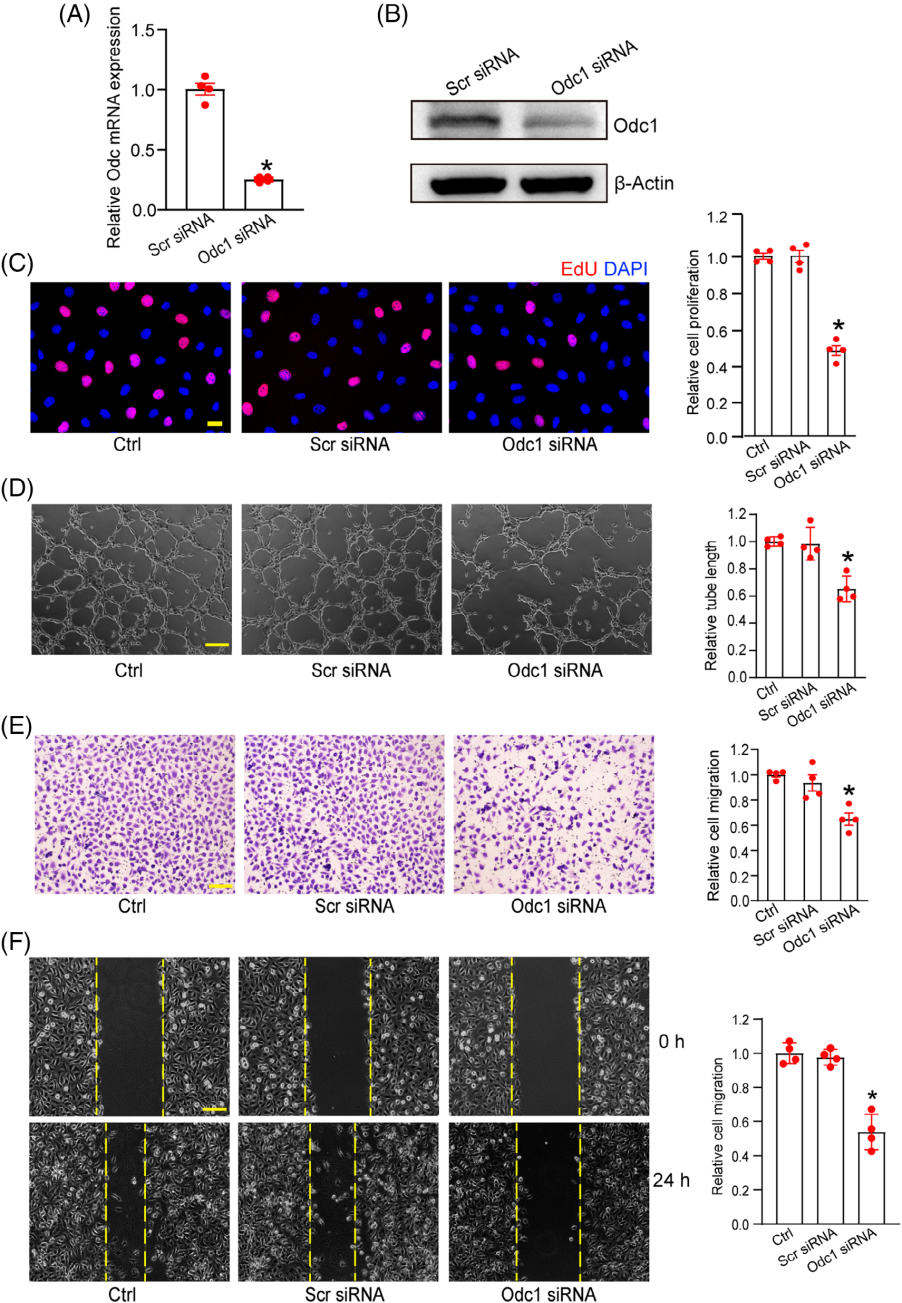
Odc1 silencing suppresses endothelial angiogenic effects in vitro. (A and B) HRMECs were transfected with Odc1 siRNA or scramble (Scr) siRNA for 24 h. Odc1 expression was detected by qRT‐PCR assays (A) and western blots (B). *n* = 4; *P < .05; Student's *t* test. (C–F) HRMECs were transfected with Odc1 siRNA or Scr siRNA, or left untreated (Ctrl) for 24 h. EdU assays were conducted to measure the proliferation of HRMECs (C, *n* = 4, scale bar: 20 µm.). Matrigel tube formation assays were conducted to evaluate tube formation ability of HRMECs (D, *n* = 4, scale bar: 50 µm). Transwell assays and wound healing assays were conducted to assess migration ability (E and F, *n* = 4, scale bar: 50 µm). *P < .05; One‐way ANOVA with the Bonferroni test. HRMEC

We next investigated the function of Odc1 in pathological angiogenesis in vivo. Intravitreal injection of Odc1 shRNA caused a marked reduction of Odc1 expression (Figure 3A,B). Odc1 inhibition by Odc1 shRNA or DFMO mitigated retinal vascular dysfunction, including decreased avascular area and decreased neovascular area in the OIR model (Figure 3C,D). Moreover, Odc1 inhibition led to reduced endothelial proliferation (Figure 3E,F). In vascular endothelial growth factor (VEGF)‐induced angiogenesis model, Odc1 inhibition alleviated VEGF‐induced vascular leakage and acellular capillary generation. Hence, inhibition of Odc1 activity alleviates retinal vascular dysfunction (Figure S6).

**FIGURE 3:**
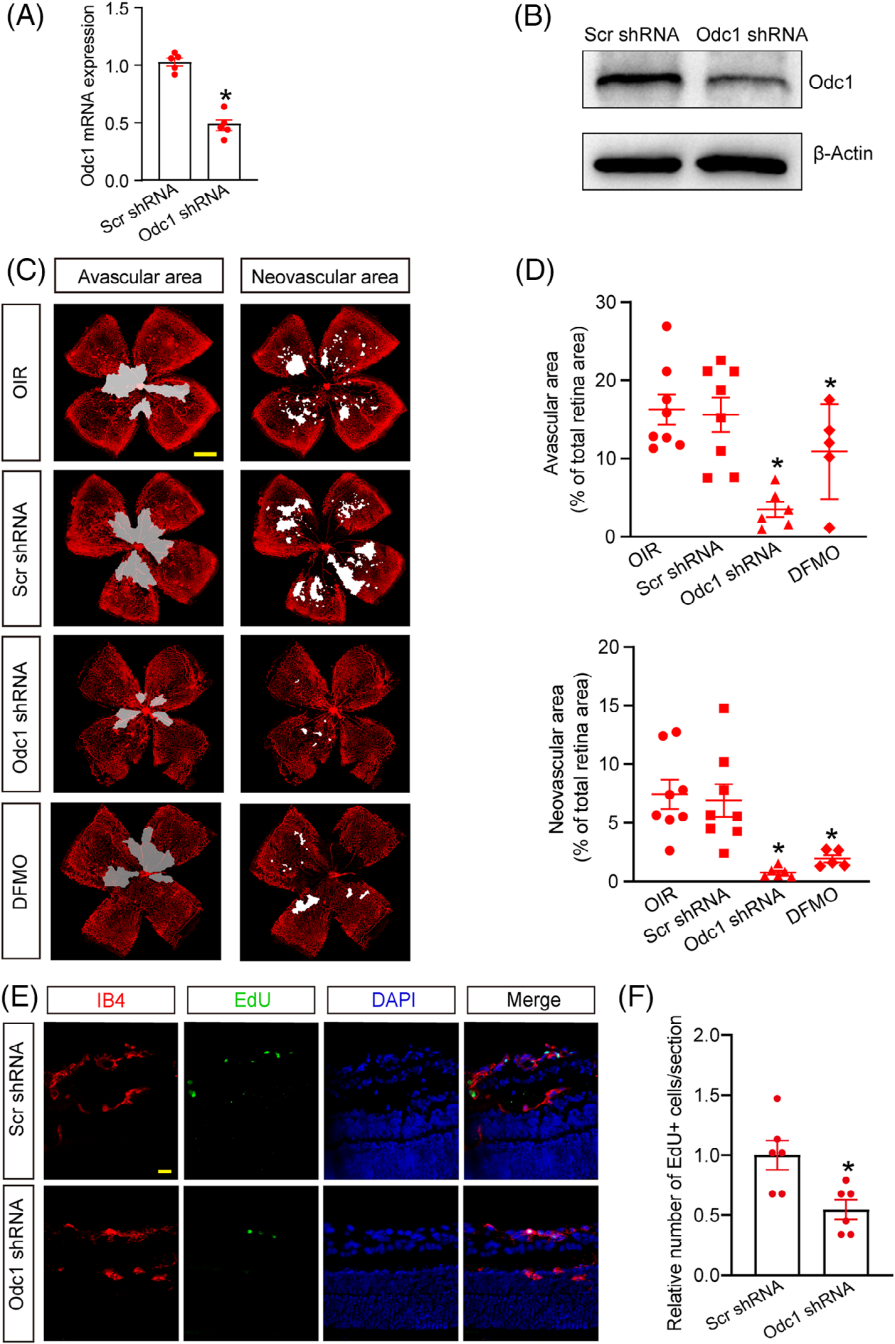
Odc1 inhibition suppresses pathological angiogenesis in vivo. (A and B) C57BL/6J mouse pups at P7 were subjected to intravitreous injections of Odc1‐specific shRNA (Odc1 shRNA) or negative control (Scr shRNA). Odc1 expression was assessed through qRT‐PCRs (A) and western blots (B). *n* = 5; *P < .05; Student's *t* test. (C and D) Representative images and quantification results of retinal avascular area and neovascular area at P17 following intravitreal injection of Odc1 shRNA or DFMO. *n* = 5–8; *P < .05; One‐way ANOVA with Bonferroni test; scale bars: 500 µm. (E) Representative images of endothelial proliferation signals in retinal sagittal sections following the transfection of Odc1 shRNA or Scr shRNA. Scale bar: 20 µm. (F) Quantitative analysis of EdU^+^ cell number. *n* = 6; *P < .05; Student's *t*‐test.

To dissect the mechanism of Odc1‐mediated angiogenesis, RNA sequencing (RNA‐seq) was conducted to assess gene expression change following Odc1 silencing. Principal component analysis (PCA) affirmed the reproducibility and reliability of the RNA‐seq datasets for subsequent analysis. Around 2 564 DEGs were identified following Odc1 silencing (Fragments per kilobase of transcript per million mapped reads (FPKM) > 1.0 and *P* < 0.05). The GO terms of DEGs were associated with angiogenic function. Gene Set Enrichment Analysis (GSEA) uncovered the involvement of cell cycle, DNA replication and aminoacyl‐tRNA biosynthesis in the Odc1‐mediated network (Figure S7).

We further conducted metabolic profiling post Odc1 silencing using mass spectrometry, identifying 274 altered metabolites (VIP > 1.0 and *P* < .05). Multi‐omics analysis was carried out by combining transcriptomic results with metabolomic results. Glutathione (GSH) metabolism emerged as the top‐ranked pathway affected by Odc1 silencing. Odc1 silencing caused a marked expression reduction of GSH metabolism‐related genes, suggesting a potential interaction between Odc1 and GSH metabolism (Figure S8).

GSH is a key member of glutathione metabolism, serving crucial functions in antioxidant defense, gene regulation and immune response.^9^ We then investigated whether exogenous GSH supplementation interrupted the inhibitory effects of Odc1 silencing on GSH metabolism. Exogenous GSH supplementation partially interrupted the anti‐angiogenic effects of Odc1 silencing in human retinal microvascular endothelial cells (HRMECs). Odc1 silencing enhanced the levels of reactive oxygen species (ROS), while GSH supplementation effectively reduced ROS levels (Figure S9). In the OIR model, Odc1 silencing alleviated retinal vascular dysfunction at P17. GSH supplementation for 4 consecutive days could interrupt the beneficial effects of Odc1 silencing and increased the area of neovascular regions. GSH supplementation contributed to EC proliferation as shown by increased EdU^+^ signal. In the VEGF‐induced angiogenesis model, GSH supplementation also interrupted the beneficial effects of Odc1 inhibition. While GSH supplementation had no discernible effects on retinal vascular leakage, it did elevate the number of acellular capillaries (Figure S10).

Finally, we explored the clinical relevance of Odc1 dysregulation in ocular vascular diseases. Aqueous humour was collected from retinopathy of prematurity (ROP) patients and age‐matched control cataract patients, revealing significantly elevated Odc1 levels in ROP patients (Figure 4A). The expression of Odc1 was diffusely distributed in fibrovascular membranes collected from ROP patients, co‐localising with retinal vessels (Figure 4B). Additionally, aqueous humour was collected from proliferative diabetic retinopathy (PDR) patients and other non‐diabetic patients.^10^ Odc1 levels were also up‐regulated in PDR patients (Figure 4C). We observed co‐localisation between Odc1 and CD31 in fibrovascular membranes collected from PDR patients (Figure 4D).

**FIGURE 4:**
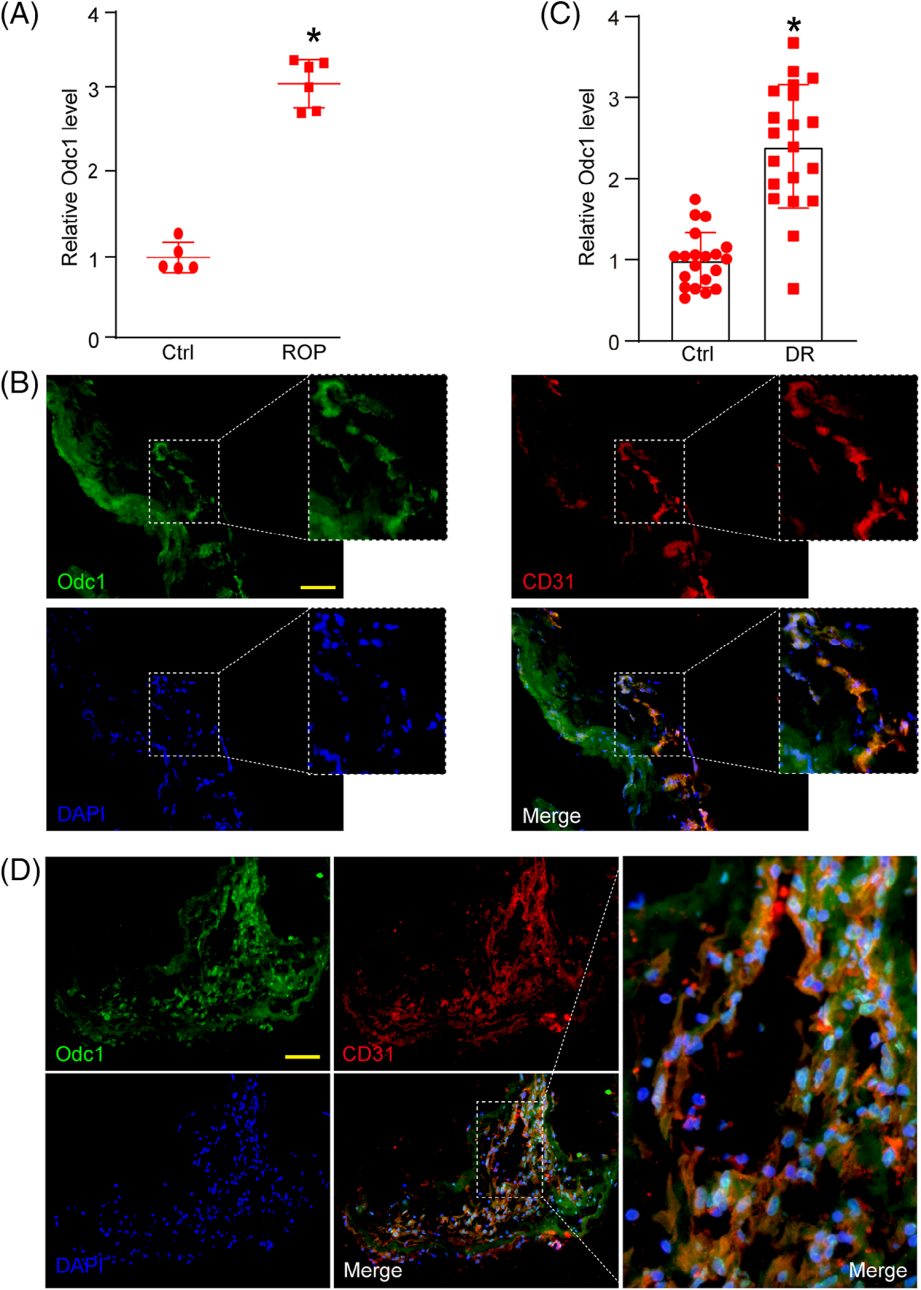
Clinical relevance of Odc1 dysregulation in retinal vascular diseases. (A) Odc1 expression in aqueous humour from the patients with ROP and age‐matched cataracts (Ctrl) was examined by enzyme‐linked immunosorbent (ELISA) assays (*n* = 5–6; *P < .05). (B) Immunofluorescence assays were conducted to detect Odc1 expression in fibrovascular membranes of ROP patients. Scale bar: 50 µm. (C) Odc1 expression in aqueous humour from the patients with PDR and age‐matched cataracts (Ctrl) was examined by ELISA assays (*n* = 20; *P < .05). (D) Immunofluorescence assays were conducted to detect Odc1 expression in fibrovascular membranes of PDR patients. Scale bar: 50 µm. PDR, proliferative diabetic retinopathy.

## CONCLUSION

Single‐cell RNA sequencing has unveiled a distinct EC subpopulation associated with pathological angiogenesis, characterised by Odc1 expression. Inhibiting Odc1 activity demonstrates pronounced anti‐angiogenic effects, mitigating pathological angiogenesis through the GSH signalling pathway. Exogenous GSH supplementation partially interrupts the anti‐angiogenic effects of Odc1 inhibition. This study presents a single‐cell atlas of EC heterogeneity during retinal angiogenesis and will open new avenues in the anti‐angiogenic treatment by targeting specific EC subpopulation.

## AUTHOR CONTRIBUTIONS

Biao Yan, Qin Jiang, and Pei‐Quan Zhao conceived and designed the study. Ming Tong, Yun Bai, Linyu Zhang, Mudi Yao, Xiaoyan Han, Xiumiao Li, and Dan Li conducted the experiments, prepared figures, and drafted the manuscript; Ming Tong, Jiao Lyu, and Ling Ren developed the methods and conducted data analysis; Ming Tong, Mudi Yao, and Biao Yan acquired and interpreted the data; Ming Tong, Ling Ren, and Biao Yan contributed to writing and revising the paper. All authors have reviewed and approved the final manuscript.

## CONFLICT OF INTEREST STATEMENT

The authors have no conflicts of interest.

## FUNDING INFORMATION

This work is supported by the grants from National Natural Science Foundation of China, Grant numbers: 82225013 and 81970809 to Dr Yan, 81570859 and 82070983 to Dr Jiang.

## ETHICS STATEMENT

All experiments adhered to the principles outlined in the Declaration of Helsinki and adhered to the ARVO statement on human subjects. The Institutional Review Board of the authors' institute granted approval for this study. All animal studies were conducted in compliance with the National Institutes of Health (NIH Publication, 8th Edition, 2011) guidelines for the use of laboratory animals and adhered to the ARVO Statement for the Use of Animals in Ophthalmic and Vision Research. The experiments were approved by the Institutional Animal Care and Use Committee of the author's institute.

## Supporting information

Supporting Information

## Data Availability

Raw metabolomic data has been deposited in MetaboLights Repository (www.ebi.ac.uk/metabolights/MTBLS7911) with Study Identifier MTBLS9397. Raw RNA sequencing data have been deposited in Genbank repository with accession number PRJNA 1066761. Raw counts matrix of single‐cell data was available on figshare at following https://doi.org/10.6084/m9.figshare.25074398.
