# Supplementary material for: Single‐cell RNA sequencing unveils an Odc1‐marked endothelial subpopulation critical for pathological angiogenesis
Source: Clin Transl Med. 2024 Mar 27;14(3):e1640. doi: 10.1002/ctm2.1640 (PMC10966522; doi:10.1002/ctm2.1640)
Supplement: Supplementary file 1 — Supporting Information [file CTM2-14-e1640-s001.docx]

**Supplemental data**

**Supplementary Figure**

**
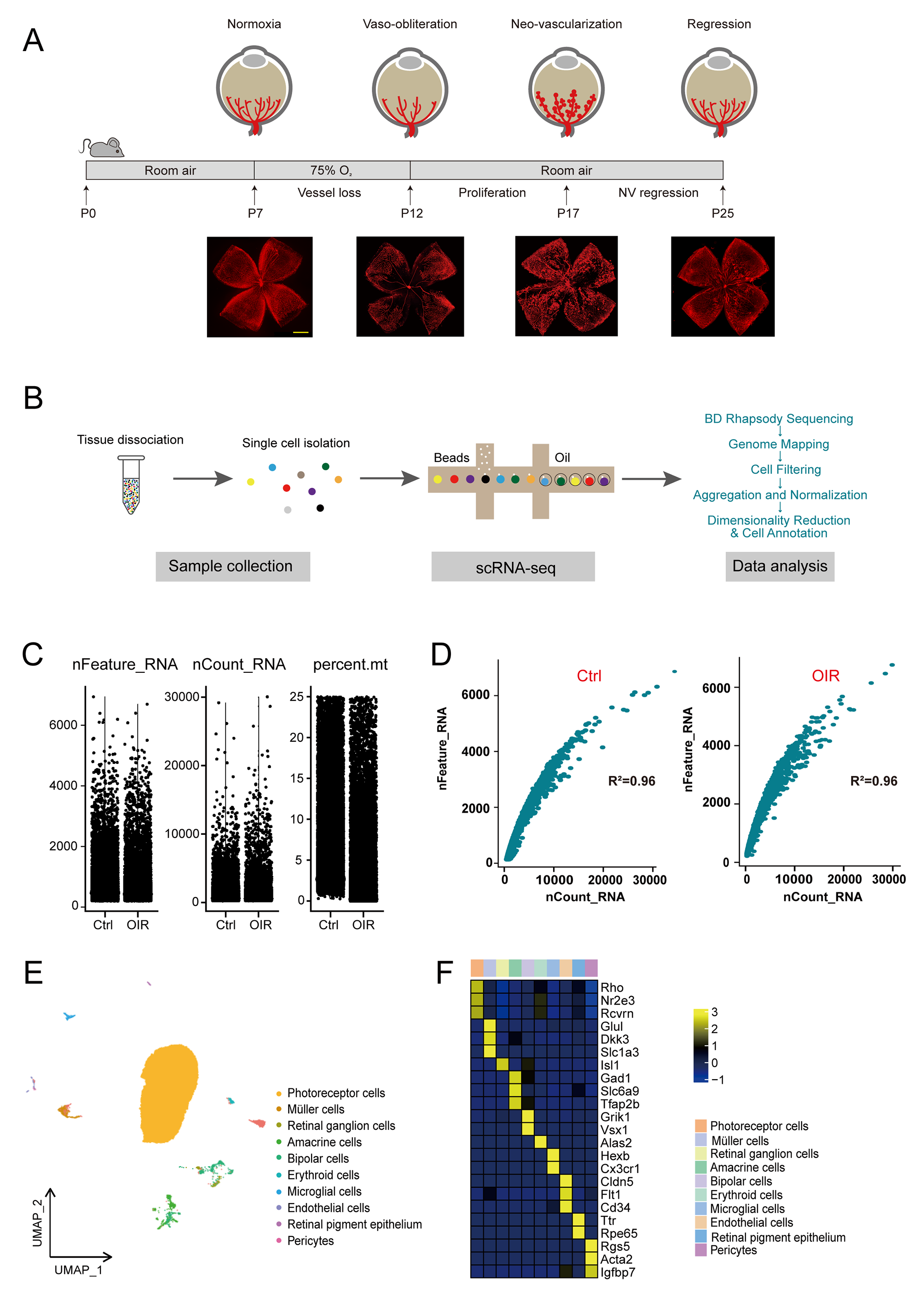
**

**Figure S1: Single-cell atlas of OIR retinas and non-OIR retinas** (A) The schematic diagram illustrates the experimental model of OIR, accompanied by representative photographs of Isolectin B4 staining depicting retinal vessels at various pathological phases. Scale bar: 500 μm. (B) The workflow for scRNA-seq of P17 control non-OIR and OIR mice retinal samples. (C) Violin plots post quality control filtering, presenting gene number per cell (nFeature_RNA), total count of all genes per cell (nCount_RNA), and the percentage of mitochondrial genes in total genes per cell (percent.mt). (D) Correlations between the number of genes per cell and the total count number of all genes in each cell using Seurat with Pearson’s Correlation. (E) Uniform manifold approximation and projection (UMAP) algorithm clustered cells into 10 types. Each color represented the annotated phenotype of each cluster. UMAP plots displaying the 10 defined cell types in OIR retinas and non-OIR retinas based on the canonical cell markers. (F) Heatmap showing the canonical markers within the defined 10 retinal cell types.


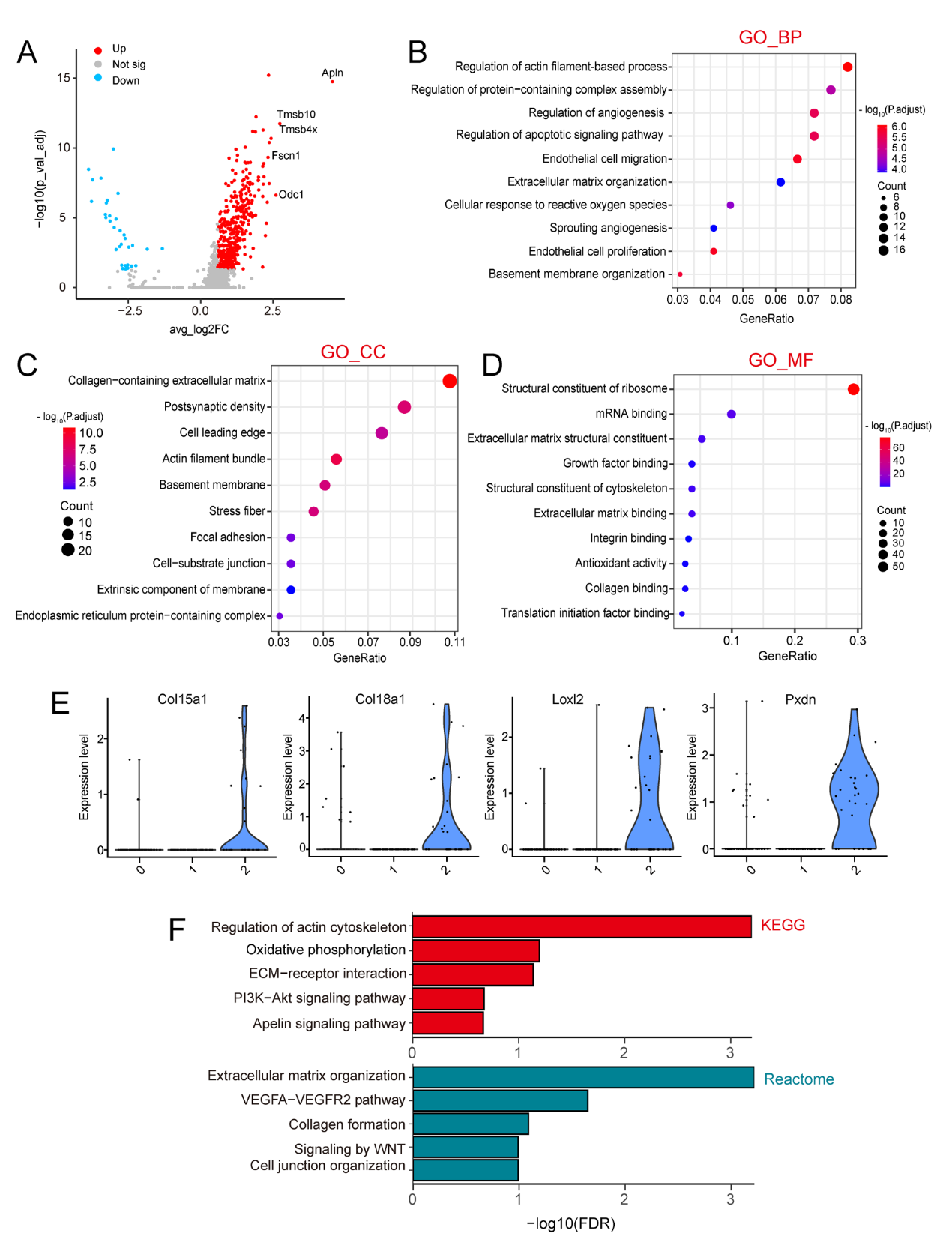


**Figure S2: Prediction of the function of EC sub-population 2** (A) Volcano plot showing DEGs between EC sub-population 2 and EC sub-population 0, 1. (B-D) GO enrichment analysis of the top 200 DEGs between sub-population 2 and sub-population 0, 1, including three GO ontologies: biological process (B), cellular component (C), and molecular function (D). (E) Violin plots showing DEGs in EC sub-population 2 related to ECM organization. (F) Bar chart showing the pathway enrichment results from 2 independent pathway databases. The lists of the top 200 DEGs between sub-population 2 and sub-population 0, 1 were used as the input for analysis.

**
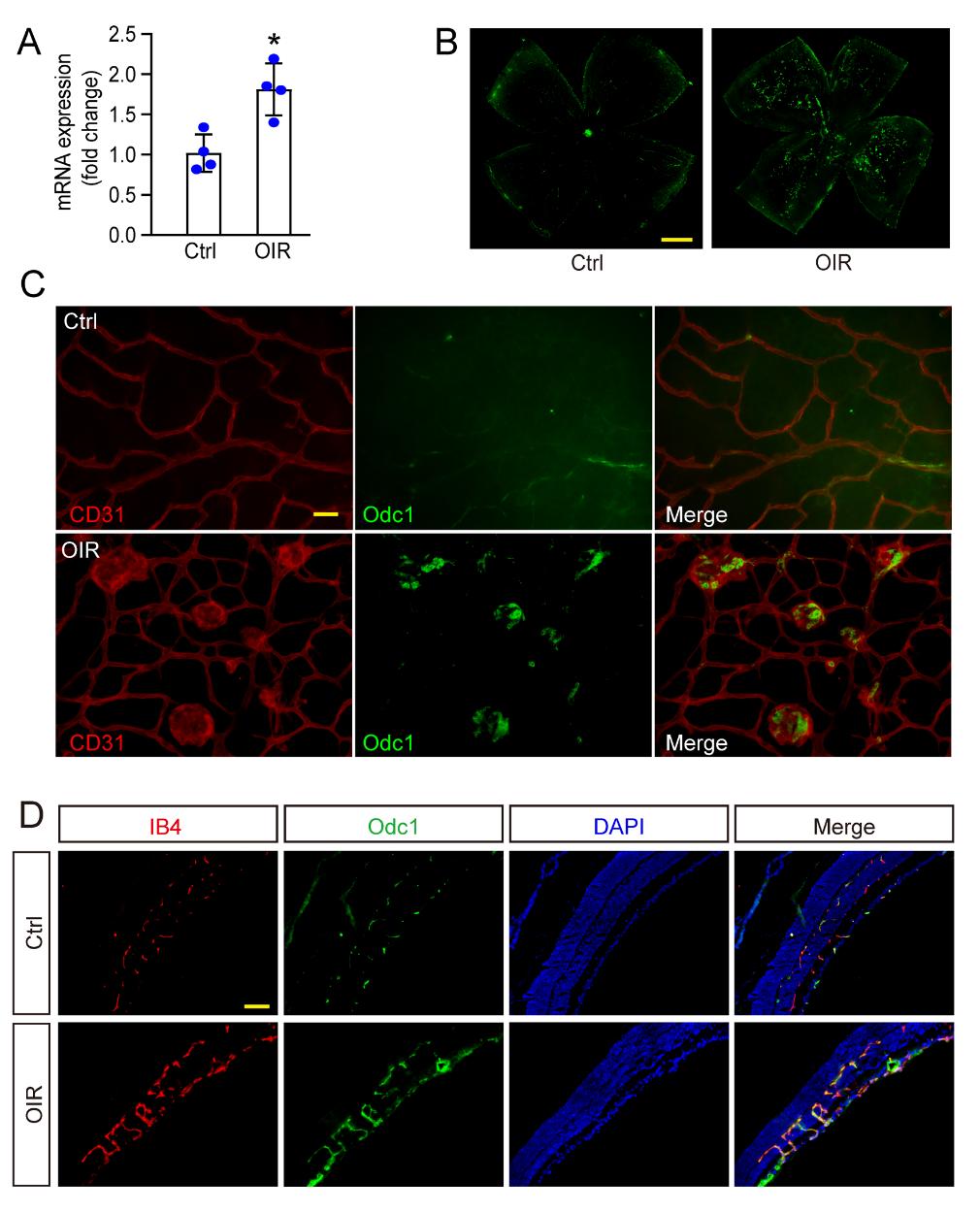
**

**Figure S3: Odc1 was up-regulated and co-localized with angiogenic region in OIR retina**

(A) qRT-PCR assays were conducted to compare the expression difference of Odc1 mRNA between OIR retinas and non-OIR control retinas at P17. Data were presented as fold change compared with non-OIR control retinas. n = 4; **P* < 0.05; Student’s *t* test. (B) Immunofluorescence assays were conducted to assess Odc1 expression in OIR retinas and non-OIR retinas at P17. Scale bar: 500 μm. (C) Representative retinal flat mounts illustrate the co-localization between retinal neovascular tufts and Odc1. CD31 was used to label retinal vessels. Scale bar: 20 μm. (D) Immunofluorescence staining was conducted to detect the co-localization between Odc1 and IB4 in retinal slices. Scale bar: 50 μm.

**
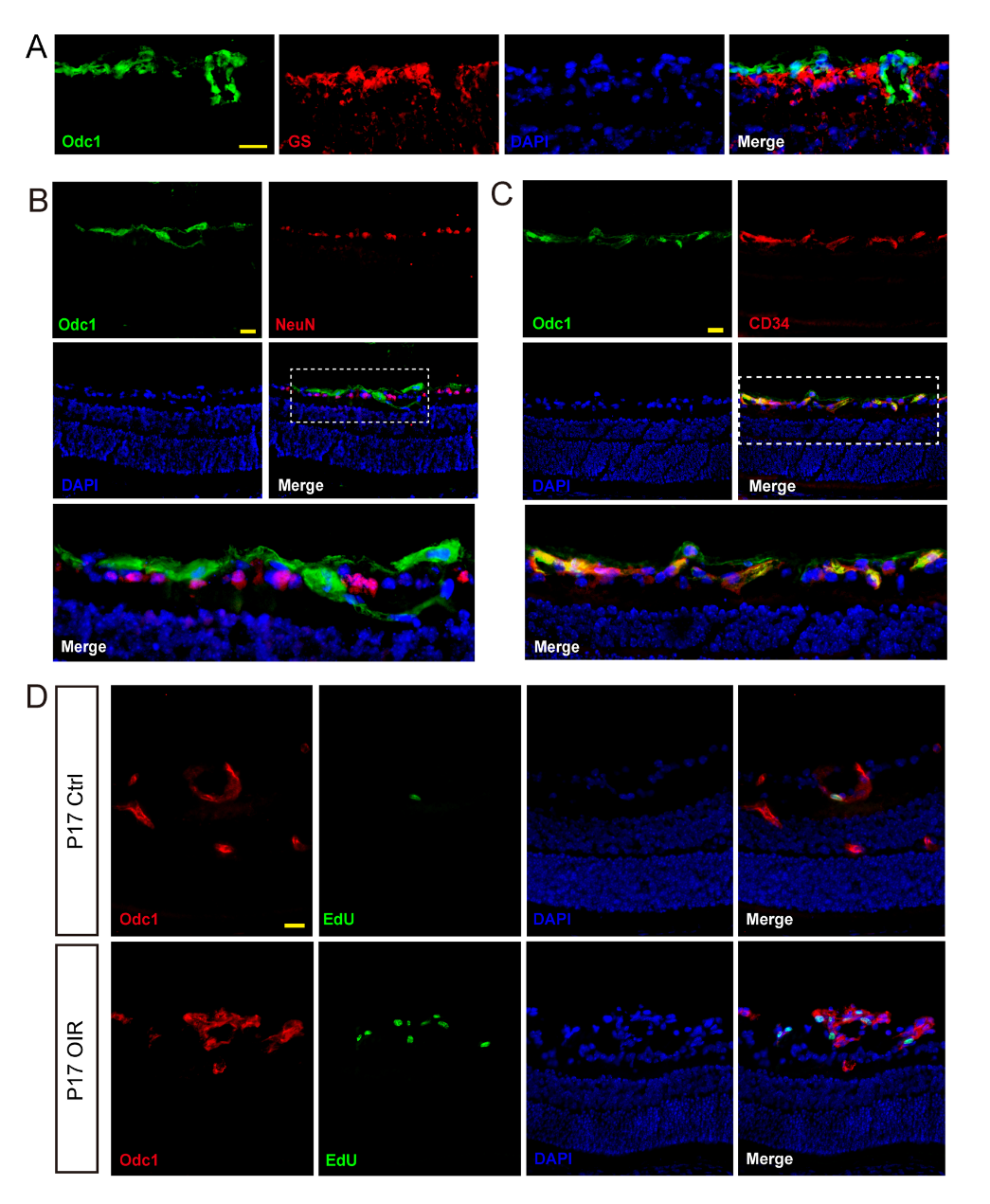
**

**Figure S4: Odc1 is specifically expressed in ECs and associated with endothelial proliferation** (A-C) Immunofluorescence assays were conducted to ascertain the co-localization between Odc1 and GS (labeling astrocytes and Müller cells, A), NeuN (labeling RGCs, B), or CD34 (labeling endothelial cells, C). Scale bar: 20 μm. (D) Representative images of proliferative signals and Odc1 staining signals in retinal sagittal sections. DAPI staining was used to label cell nuclei. Scale bar: 20 μm.

**
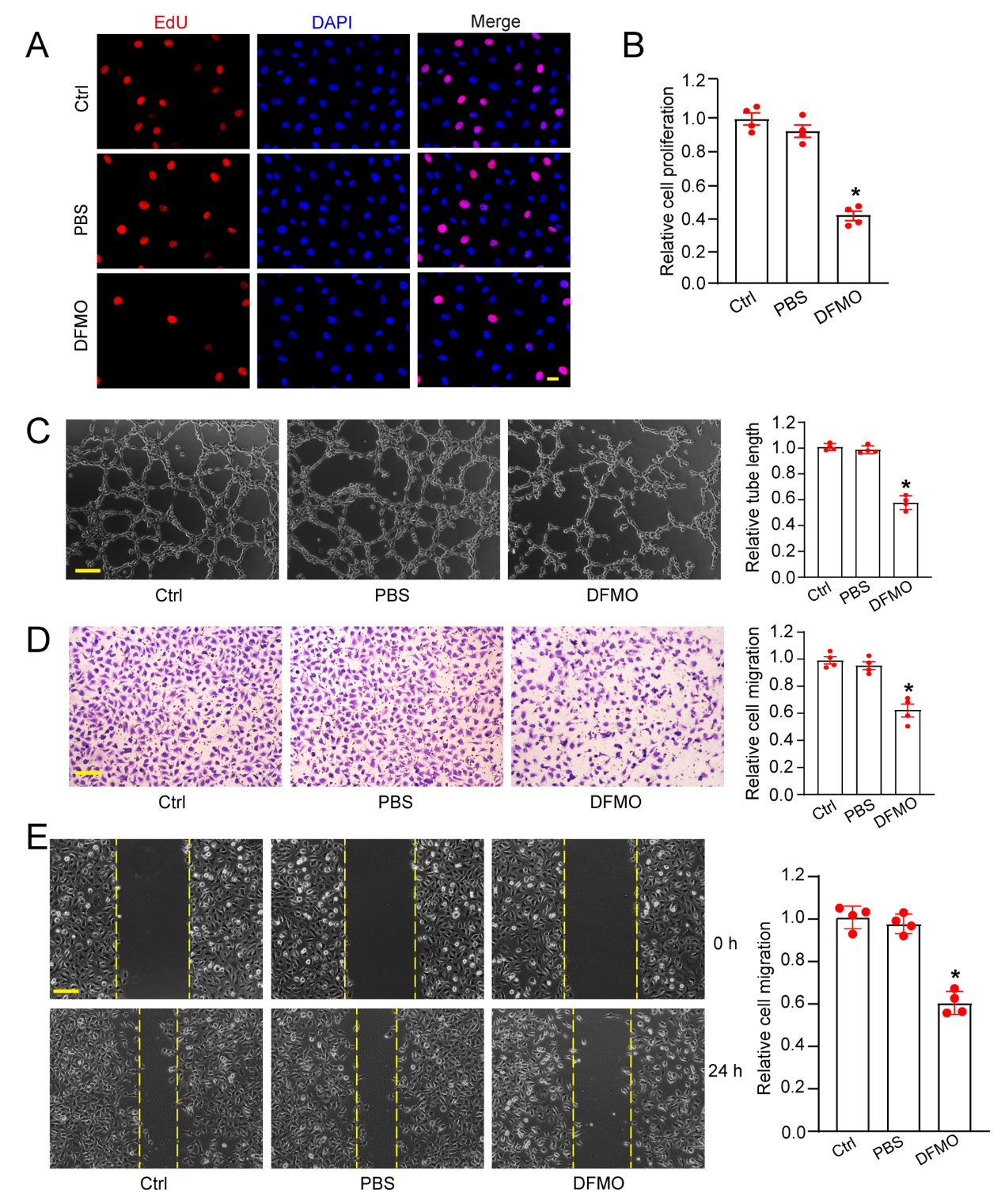
**

**Figure S5: Odc1 inhibitor suppresses endothelial angiogenic effects *in vitro***

(A and B) HRMECs were treated with DFMO (500 μM), PBS, or left untreated (Ctrl) for 24 h. EdU staining assays were conducted to detect the proliferation of HRMECs. Scale bar: 20 μm. (C) Matrigel tube formation assays were conducted to detect tube formation ability of HRMECs. Scale bar: 50 μm. (D) Transwell assays and wound healing assays were conducted to detect migration ability of HRMECs. Scale bar: 50 μm. n = 4; **P* < 0.05; One-way ANOVA with Bonferroni test.

**
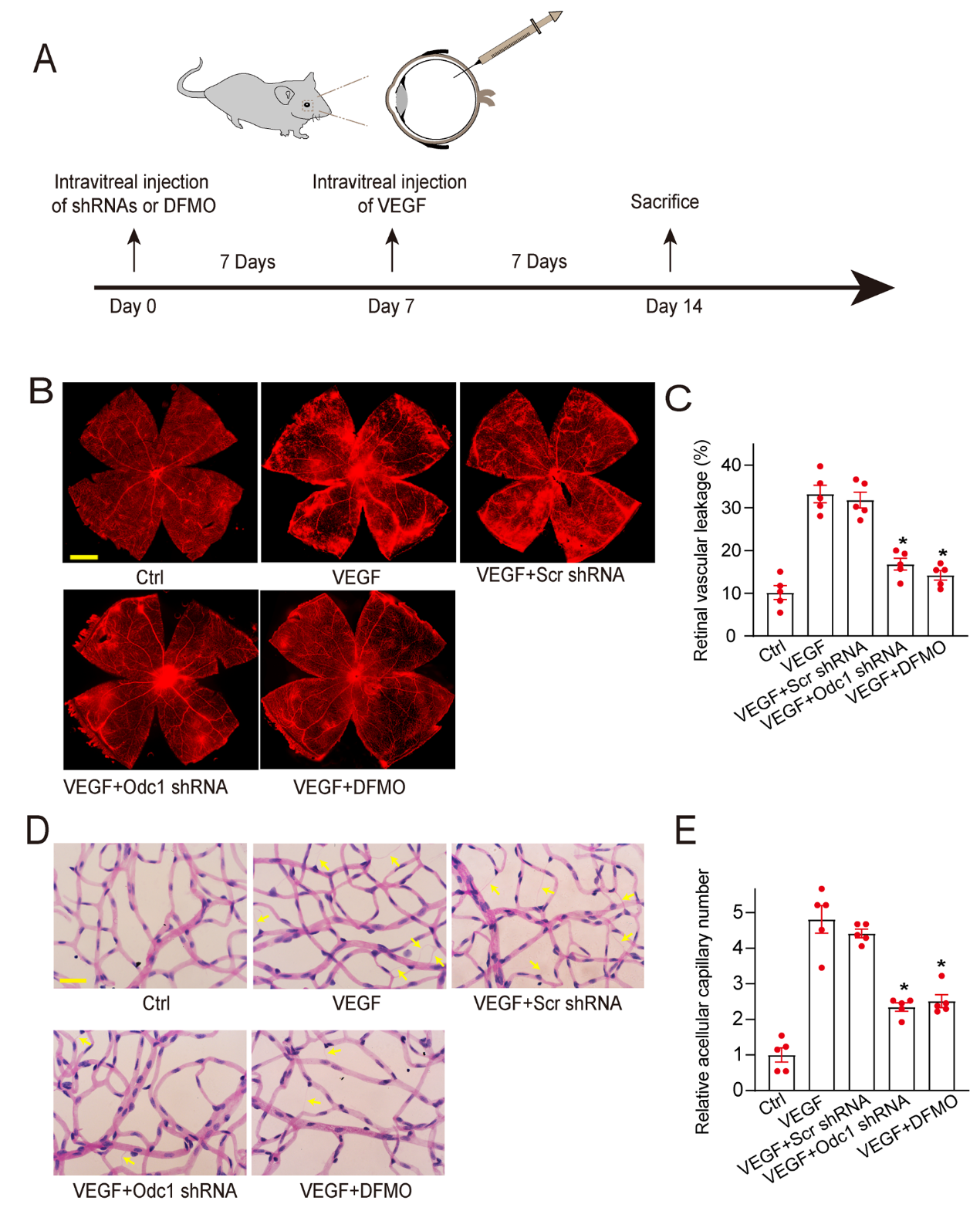
**

**Figure S6: Odc1 inhibition alleviates VEGF-induced retinal vascular dysfunction**

(A) On day 7 following intravitreal administration of Odc1 shRNA or DFMO, C57BL/6J mice underwent intravitreal injection of VEGF to initiate the development of an ocular angiogenesis model. (B and C) Evans blue assays were conducted to detect retinal vascular leakage. Scale bar: 500 μm. n = 5; **P* < 0.05; One-way ANOVA with Bonferroni test. (D and E) Trypsin digest and periodic acid Schiff (PAS) staining was conducted to detect retinal acellular capillaries. Scale bar: 10 μm. n = 6; **P* < 0.05; One-way ANOVA with Bonferroni test.

**
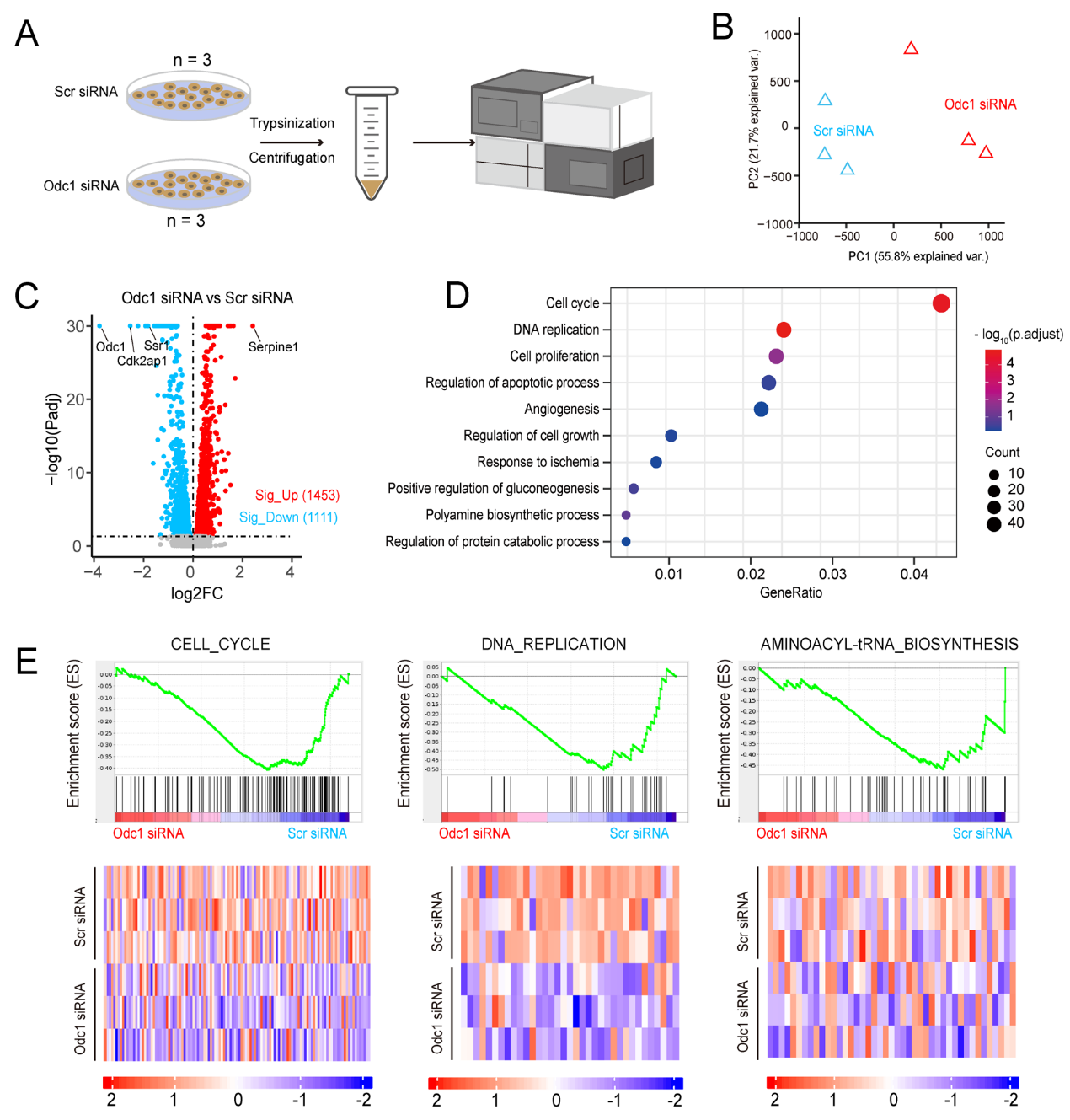
**

**Figure S7: Transcriptomic profiling reveals gene expression change in HRMECs following Odc1 silencing**

(A) Schematic illustration of transcriptomic profiling of HRMECs transfected with Odc1 siRNA or Scr siRNA (n = 3). (B) PCA of RNA-seq data from HRMECs transfected with Odc1 siRNA and Scr siRNA (n = 3). Each replicate was shown as a single triangle. (C) Volcano plots showing DEGs between Odc1 siRNA group and Scr siRNA group. (D) GO_BP enrichment analysis of DEGs between Odc1 siRNA group and Scr siRNA group. (E) Representative GSEA and corresponding heatmap plots of associated genes between Odc1 siRNA group and Scr siRNA group. Heatmap plots showing cell cycle, DNA replication and aminoacyl-tRNA biosynthesis pathway down-regulated in Odc1 siRNA group.

**
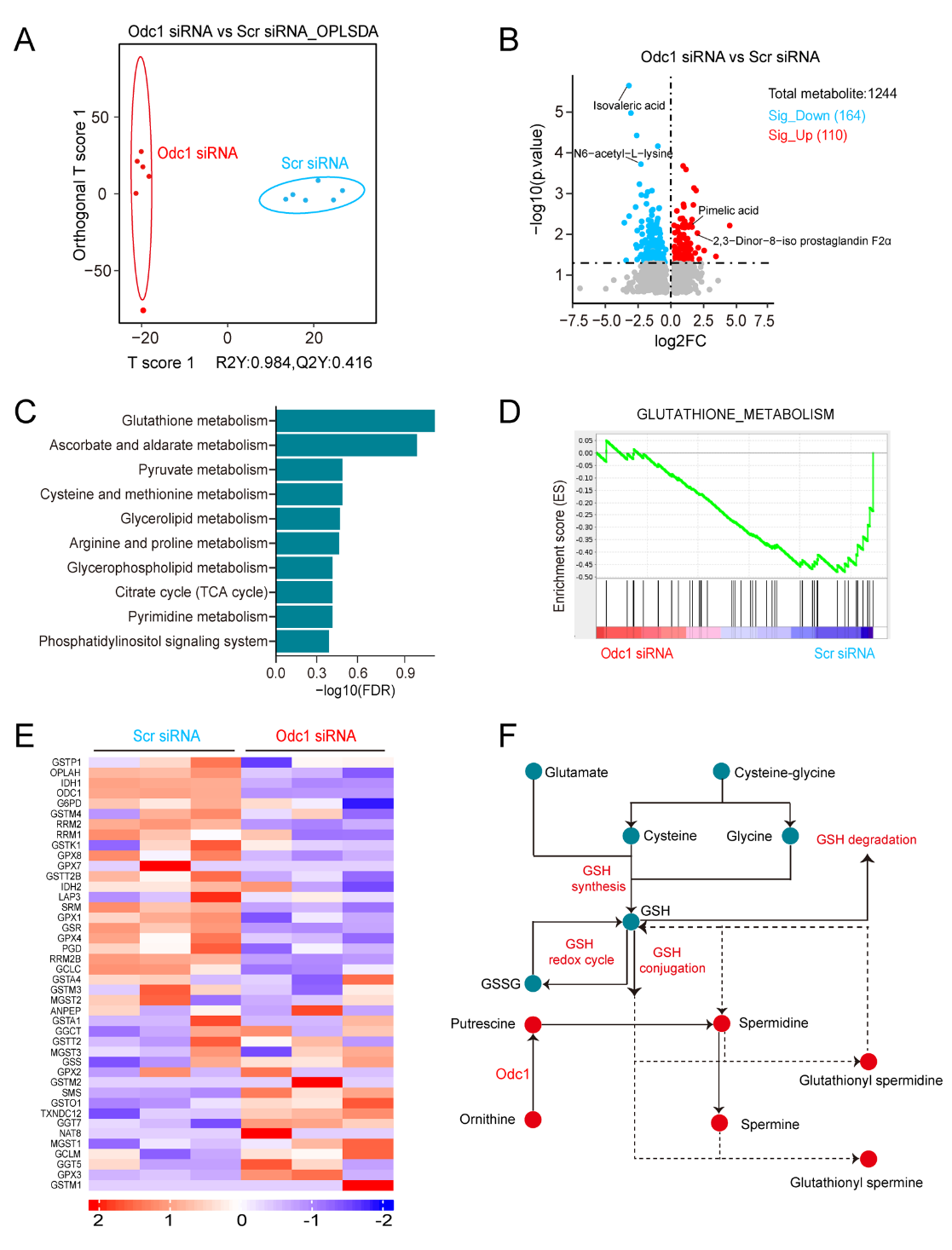
**

**Figure S8: Metabolic profiling identifies altered GSH metabolism in HRMECs following Odc1 silencing**

(A) Metabolite profiles of HRMECs transfected with either Odc1 siRNAs or Scr siRNAs (n = 6) were analyzed using Orthogonal Partial Least Square Discriminant Analysis (OPLS-DA). (B) Volcano plots illustrate the differential metabolites between the Odc1 siRNA group and Scr siRNA group. (C) Multi-omics analysis integrating transcriptomic data and metabolomics data was conducted using the Joint Pathway Analysis (JPA) module of MetaboAnalyst 5.0. The top 10 enriched pathways were presented. (D and E) Gene set enrichment analysis (GSEA) and heatmap analysis showcased the expression pattern of genes related to glutathione metabolism. (F) The pathway diagram depicted the interaction between GSH metabolism and Odc1-mediated polyamine synthesis metabolism. The circles represent metabolites.

**
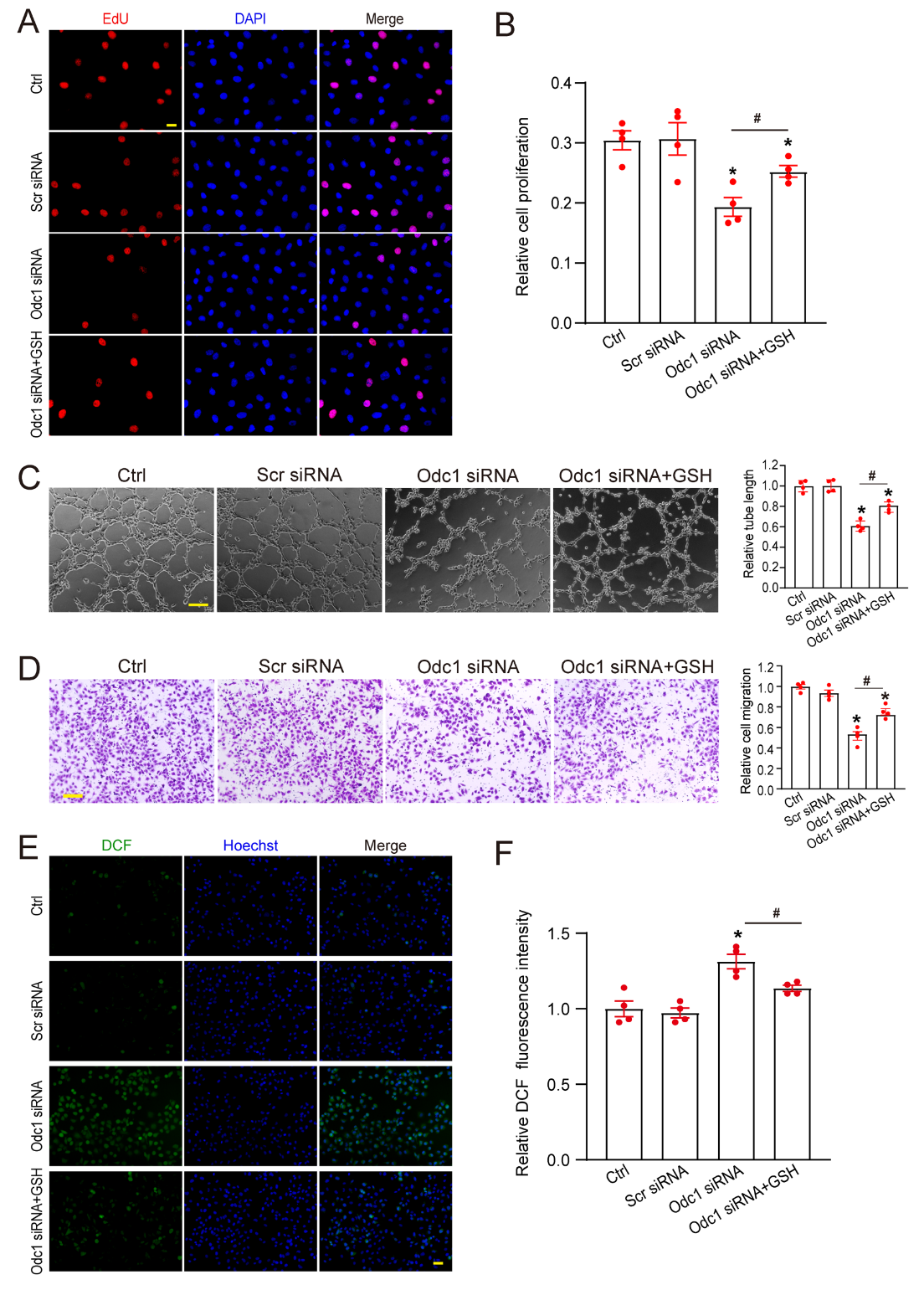
**

**Figure S9: GSH supplementation interrupts the anti-angiogenic effects of Odc1 silencing *in vitro***

(A and B) HRMECs were transfected with Odc1 siRNA, scramble (Scr) siRNA, Odc1 siRNA plus GSH (20 mM) or left untreated (Ctrl) for 24 h. EdU assays were conducted to detect the proliferation of HRMECs. Scale bar: 20 μm. (C) Matrigel assays were conducted to detect the tube formation of HRMECs. Scale bar: 50 μm. (D) Transwell assays were conducted to detect the migration ability of HRMECs. Scale bar: 50 μm. (E and F) ROS assays were conducted to assess the levels of ROS in HRMECs. Scale bar: 50 μm. n = 4; **P* < 0.05; ^#^*P* < 0.05; One-way ANOVA with Bonferroni test.

**
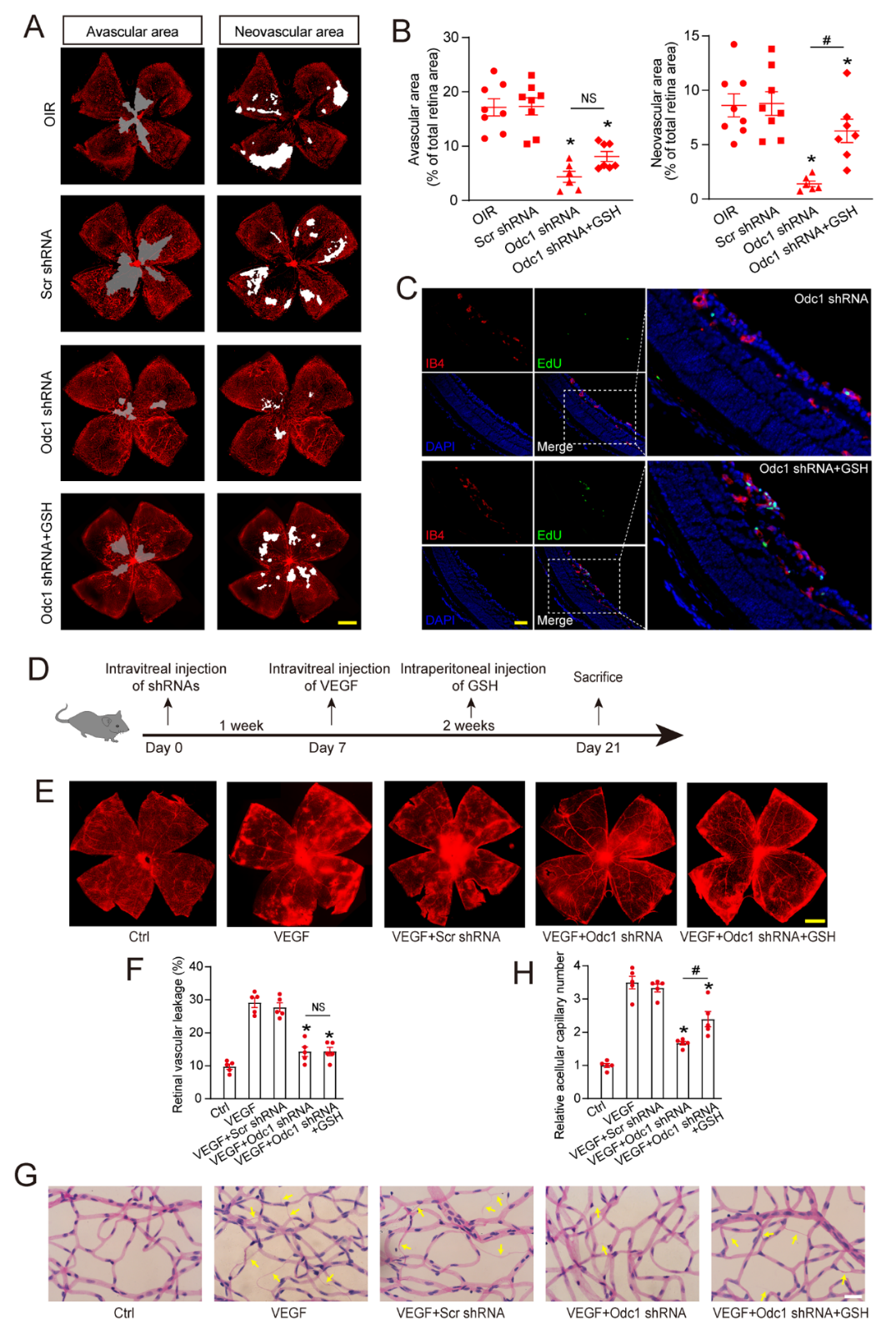
**

**Figure S10: GSH supplementation interrupts the anti-angiogenic effects of Odc1 silencing**

(A and B) Representative images and quantification results of retinal avascular areas and neovascular areas at P17 in OIR mice with or without intraperitoneal injection of GSH following Odc1 silencing. n = 6 - 8; **P* < 0.05; ^#^*P* < 0.05; One-way ANOVA with Bonferroni test; Scale bar: 500 μm. (C) Representative images of endothelial proliferation signals in retinal sagittal sections with or without intraperitoneal injection of GSH following Odc1 silencing. Scale bar: 50 μm. (D) Time course of VEGF-induced ocular angiogenesis model. GSH was administered intraperitoneally for 2 weeks. (E and F) Evans blue assays were conducted to detect retinal vascular leakage. Scale bar: 500 μm. (G and H) PAS staining was conducted to detect retinal acellular capillaries. Scale bar: 10 μm. n = 5; **P* < 0.05; ^#^*P* < 0.05; NS, not significant; One-way ANOVA with Bonferroni test.

**Table S1: Antibodies used in this study**

| Name | Company | Host | Catalog# | Dilution | Applications |
| --- | --- | --- | --- | --- | --- |
| Odc1 | Abcam | Mouse | ab193338 | 1:200 | IF |
| GS | Abcam | Rabbit | ab176562 | 1:500 | IF |
| CD31 | Abcam | Rabbit | ab182981 | 1:50-1:100 | IF |
| CD34 | Abcam | Rabbit | ab81289 | 1:50-1:100 | IF |
| NeuN | Abcam | Rabbit | ab177487 | 1:200 | IF |
| Isolectin  GS-IB4 | Invitrogen | Alexa Fluor™  594 Conjugate | I21413 | 1:100 | IF |
| Secondary  antibodies | Invitrogen | Alexa Fluor™  488 Conjugate | A-11001 | 1:500 | IF |
| Secondary  antibodies | Invitrogen | Alexa Fluor™  594 Conjugate | A-11012 | 1:500 | IF |
| Odc1 | Abcam | Mouse | ab193338 | 1:1500 | WB |
| β-actin | ZenBio Antibody | Rabbit | 380624 | 1:1000 | WB |
| IgG (H+L) | Beyotime | Mouse | A0216 | 1:1000 | WB |
| IgG (H+L) | Beyotime | Rabbit | A0208 | 1:1000 | WB |

**Table S2: Primer sequences used for qRT-PCR assays**

| **Species** | **Gene** | **Sequences (5’ to 3’)** |
| --- | --- | --- |
| Human | Odc1 | Forward: CTGGGCGCTCTGAGATTGTC |
|  |  | Reverse: TGGTCCAGAATGTCCTTGGC |
| Human | β-Actin | Forward: TTGTTACAGGAAGTCCCTTGCC |
|  |  | Reverse: ATGCTATCACCTCCCCTGTGTG |
| Mouse | Odc1 | Forward: GGCTAAGTCGACCTTGTGAGG |
|  |  | Reverse: GGCTAAGTCGACCTTGTGAGG |
| Mouse | β-Actin | Forward: GTGGATCAGCAAGCAGGAGTA |
|  |  | Reverse: GTGTAAAACCCAGCTCAGTAACA |

**Materials and methods**

**Animal experiment**

C57BL/6J mice were purchased from Hangzhou Ziyuan Laboratory Animal Technology Co., Ltd. (Hangzhou, China). The animals were housed in a controlled environment with stable conditions of light and temperature (12 h cycles of light-darkness, 18-24℃), allowing for free access to water and food. All animal studies were conducted in compliance with the National Institutes of Health (NIH Publication, 8th Edition, 2011) guidelines for the use of laboratory animals and adhered to the ARVO Statement for the Use of Animals in Ophthalmic and Vision Research. All experiments were approved by the Institutional Animal Care and Use Committee of the author’s institute.

**Oxygen-induced retinopathy (OIR) model**

C57BL/6J mouse pups, including both males and females, along with their nursing mothers, were subjected to 75% O2 (hyperoxia) from postnatal day 7 (P7) to P12 and then returned to room air (normoxia). Hyperoxia induced oxygen toxicity and reduced the levels of angiogenic mediators, which led to vaso-obliteration. The transition to a relatively hypoxic environment from P12 facilitated the development of pathological neovascularization. At P17, the retinas of these mouse pups were harvested. To detect endothelial proliferation, 5-ethynyl-2’-deoxyuridine (EdU) (6 μg/ul, Beyotime Biotechnology, Shanghai, China) was intraperitoneally injected into P17 neonatal mice 4 h before they were sacrificed.

**Periodic Acid-Schiff's reagent (PAS) assay**

To assess changes in retinal vasculature, the mice were sacrificed and their eyes were enucleated and fixed in 10% neutral formaldehyde for 24 h. The retinas were then isolated and rinsed in double distilled water overnight. Subsequently, the retinas were incubated with trypsin (BioFroxx, Germany) at 37℃ for 1 h until flocculent tissues appeared. Excess retinal tissues were gently flushed away using a 1 ml syringe filled with PBS. The remaining vascular network were carefully mounted on glass slides for dry. Afterwards, all retinas were stained using a Periodic Acid Schiff (PAS) Stain Kit according to the manufacturer's instruction (Solarbio, China). An inverted light microscope (Olympus, Japan) was used to observe the structure changes of retinal capillaries and the number of acellular capillaries was counted.

**Evans blue (EB) assay**

EB assays were used to detect retinal vascular leakage. EB dye (KEHBIO Technology, Beijing, China) was prepared by dissolving in saline (30 mg/ml) on the shaker table overnight. The solution was then filtered through a 0.45-µm filter. Following anesthesia, the internal skin of one thigh was incised and the soft tissues were carefully separated to expose the femoral vein. EB dye was injected into the femoral vein using an insulin syringe (KINDLY, shanghai, China) at the dose of 45 mg/kg. The mice were then placed on a rewarming table for about 30 min to facilitate circulation. Afterward, their eyeballs were enucleated and fixed in 4% paraformaldehyde (PFA) for 50 min. Finally, the retinas were mounted and examined for vascular leakage under a fluorescence microscope (Olympus, Japan).

**Single-cell sequencing and analysis**

To capture the cellular diversity in the retinas, four retinas from OIR group or non-OIR group at P17 were harvested and pooled to create a single sample. Subsequently, the samples representing each group were processed for single-cell sequencing. The enzyme digestion was applied to dissociate the retinas into the single-cell solution. The resulting single-cell suspension was then processed using the BD Rhapsody system. Single-cell transcriptomes captured by microbeads were transformed into cDNA libraries containing cell labels and unique molecular identifiers (UMIs). The libraries were sequenced in a PE150 mode (Pair-End for 150 bp read) on the 10X instrument (Illumina).

Raw reads were subjected to the BD Rhapsody Whole Transcriptome Assay Analysis Pipeline (Early access) for further processing, including quality filtering, read and molecule annotation, cell determination, and the creation of single-cell expression matrices. The output matrices were then corrected using RSEC and DBEC algorithms.

Raw gene expression matrices were separately imported into R (version 4.2.2) and transformed into the Seurat objects using the Seurat R package (version 4.3.0). The gene expression matrix was then normalized to total cellular UMI count. Normalization was performed based on the total cellular UMI count. Gene features expressed in at least three cells and cells with a detection of at least 200 genes were retained. Cells with fewer than 200 or more than 8000 gene features, as well as those with mitochondrial counts exceeding 25%, were filtered out. The top 2000 features were selected as highly variable genes for subsequent clustering analysis.

After scaling the data with respect to UMI counts, principal components analysis (PCA) was applied to reduce dimensionality using the highly variable genes identified earlier. The Harmonypackage was used to remove the batch effects between the two samples. Subsequently, the Uniform Manifold Approximation and Projection (UMAP) was employed to visualize retinal cell clusters. Each cluster was identified by “FindAllMarkers” (min.pct = 0.25, logfc.threshold = 0.25, only.pos = TRUE) function of Seurat, with marker genes considered as differentially expressed genes (DEGs) for further analysis. The top 200 DEGs were selected for pathway enrichment analysis and Gene Ontology (GO) analysis, including biological process (BP), cellular component (CC), and molecular function (MF).

**Cell culture and transfection**

Human retinal microvascular endothelial cells (HRMECs) were purchased from Tongpai Biotechnology Co. Ltd (Shanghai, China). They were cultured in Endothelial Cell Medium (ECM) (Sciencell, USA) with 10% fetal bovine serum (FBS) and 20 μg/ml endothelial cell growth supplement (ECGS) at 37℃ with 5% CO_2_. The media were changed every 24 h. When HRMECs reached approximately 70% confluence, the cells were treated with Eflornithine hydrochloride hydrate (DFMO, MCE, USA) to inhibit Odc1 activity. For *in vitro* Odc1 silencing, small-interfering RNAs (siRNAs) targeting Odc1 (Odc1 siRNA) and a negative control (Scr siRNA) were purchased from Ribobio (Guangzhou, China) and transfected into HRMECs using lipofectamine 6000 (Beyotime Biotechnology, China). The silencing efficiency of Odc1 siRNA was confirmed by western blots and qRT-PCR assays.

**Clinical sample collection**

All experiments adhered to the principles outlined in the Declaration of Helsinki and adhered to the ARVO statement on human subjects. The Institutional Review Board of the authors' institute granted approval for this study. Aqueous humor was collected from the patients with ROP and age-matched control patients with cataracts. The expression levels of Odc1 in these samples were evaluated using ELISA. Each sample was prepared with three replicates and the average value from multiple wells was used as the measurement value for one sample. Pre-retinal fibrovascular membrane and proliferative membrane samples were obtained with the informed consent of ROP and PDR patients, respectively. These membrane samples were cut into 10 µm-thickness sagittal sections for immunofluorescence staining after 4% PFA fixation, sucrose dehydration, and Opti-mum Cutting Temperature (OCT) gel embedding.

**Intravitreal injection**

In the OIR model, pup mice received intravitreal injections of 1.5 µl lentivirus particles containing either 5×10^8^ TU/ml of Odc1 shRNA or negative control sequence (Scr shRNA) prior to their exposure to hyperoxia. Intravitreal injection was performed under a dissecting microscope using a pulled-glass micropipette (Hamilton Co., Reno, neovascularization) with a 33-gauge needle. The injection site was located just posterior to the limbus, penetrating the sclera, and entering the vitreous cavity. All injection sites were consistently positioned just posterior to the limbus, penetrating the sclera, and entering the vitreous cavity.

For adult C57BL/6 mice, they were anesthetized and administrated intravitreally with DFMO (5 mM), Odc1 shRNA or Scr shRNA (2.5 µl per eye, injection within 20 s) under a dissecting microscope. VEGF (10 µg/ml) intravitreal injection was performed one week after Odc1 shRNA intervention. The mice were observed carefully throughout experimental period and eyes with lens injury, vitreous hemorrhage, or intraocular inflammation were excluded.

**Immunolabeling of retinal flat-mounts**

The mice pups were sacrificed at P17 and the enucleated eyes were fixed in 4% PFA for 30 min at room temperature. Subsequently, retinas were carefully isolated, and then blocked and permeabilized with PBS containing 5% BSA and 1% TritonX-100 for 1 h at 37℃. For the visualization of angiogenic areas and avascular areas, the flat-mounted retinas were stained with isolectin-B4 (IB4, 1:50, Invitrogen) overnight at 4℃. For immunofluorescence staining of Odc1 in the retina, anti-Odc1 antibody (1:200, Abcam, UK) was used to stain retinal tissue overnight at 4℃. Retina flat-mounts were observed under a microscope (Olympus, Japan) and analyzed using ImageJ software.

**Immunostaining of retinal sagittal section**

Enucleated eyes were fixed in 4% PFA for 12 h at room temperature and subsequently immersed in 30% sucrose solution for 36 h. Following embedding in the OCT compound and snap-frozen, the eye globes were cut into 10 µm-thickness sagittal slices. Subsequently, the sections were blocked and permeabilized with a solution containing 5% BSA and 1% TritonX-100 for 1 h at 37℃. Subsequently, primary antibodies (Supplementary Table S1) were diluted and incubated with the slices overnight at 4℃. After washing three times with PBST, the slices were incubated with the fluorescence-conjugated secondary antibody for 2 h at room temperature. The nuclei were counterstained with DAPI (1:5000 in PBS). Finally, retinal slices were mounted and observed under a microscope (Olympus, Japan).

**Quantification of retinal avascular and neovascular areas**

The whole-mount retinal images were captured at ×4 magnification using a fluorescence microscope. Subsequently, these images were imported into Adobe Photoshop 2021 for the purpose of creating a comprehensive retina image. To analyze the avascular areas, we employed the paint bucket tool to select and highlight the delineated avascular regions. To assess neovascular (NV) areas, the 'magic wand tool' was used with a carefully set tolerance to identify NV areas while excluding normal vessels. Finally, Image J was employed to compute the avascular areas, NV areas, and the overall retinal areas. A comparative analysis was also conducted, contrasting the avascular and NV areas against the total retinal areas.

**RT-qPCR**

Total RNAs were isolated from HRMECs or retinal tissues using a FastPure Cell/Tissue Total RNA Isolation Kit V2 (RC112-01, Vazyme, Nanjing, China) following the manufacturer's instructions. Then, the RNAs were reversely transcribed into cDNAs using the HiScript II Q RT SuperMix for qPCR (R223-01, Vazyme, Nanjing, China). The ChamQ SYBR qPCR Master Mix (Q321-02, Vazyme, Nanjing, China) was applied for RT-qPCR assays. Relative expression changes of target genes were calculated by 2^-ΔΔCt^ method. The sequences of gene-specific primers were listed in Supplementary Table 2.

**Western blot**

The proteins from retinal tissues and HRMECs were extracted using the lysis buffer (RIPA, Beyotime Biotechnology, Shanghai, China) supplemented with the protease inhibitors. The protein concentration was determined using a bicinchoninic acid (BCA) protein assay kit (Thermo Fisher, California, USA). Equal amounts of protein were subjected for 10% SDS-PAGE electrophoresis and transferred onto the nitrocellulose membranes. The membranes were blocked with 5% skim milk solution for 2 h at room temperature and then incubated with the primary antibodies (Supplementary Table 1) overnight at 4 ℃. On the following day, anti-mouse or anti-rabbit immunoglobulin G (IgG) secondary antibodies were applied. The protein bands were visualized using Chemiluminescent Substrate (P10100, NCM Biotech, Suzhou, China) and the band intensities were quantified using ImageJ software.

**Transcriptomics profiling**

HRMECs were transfected with Odc1 siRNA or scramble siRNA for 24 h. Total RNAs were extracted for subsequent high-output RNA-Seq profiling analysis (n=3 per group). NanoDrop 2000 Spectrophotometer (Thermo Fisher Scientific) was used to detect the concentration and purity of RNA samples and the Agilent 2100 Bioanalyzer and 2100 RNA Nano 6000 Assay Kit (Agilent Technologies) was used to detect the integrity of RNA samples. Following quality control, cDNA libraries were constructed and Illumina platform was used for RNA sequencing. Raw data underwent quality control were aligned to reference genome to locate the sequencing reads. The read counts of transcripts were calculated and the expression levels of different genes were quantified using FPKM. After paired comparison of identified genes, the transcriptomic data were analyzed to sort out the differentially expressed genes (DEGs) with the threshold at FPKM ≥ 1 and adjusted *P* value < 0.05. These DEGs were used for subsequent GO and KEGG enrichment analysis based on the DAVID website (https://david.ncifcrf.gov/).

**Untargeted metabolomics profiling**

HRMECs, transfected with Odc1 siRNA or Scramble siRNA, were harvested via trypsinization (n = 6 per group). Metabolites were extracted and chromatographic fractionation of the target compounds was conducted by Vanquish (Thermo Fisher Scientific) Ultra High Performance Liquid Chromatography (2.1 mm × 100 mm, 1.7 μm) with Waters ACQUITY UPLC BEH Amide (2.1 mm × 100 mm, 1.7 μm) liquid chromatography column. The sample disk temperature was 4 ℃ and the injection volume was set as 2 μl. Primary and secondary mass spectrometry data were collected using the Thermo Q Active HFX mass spectrometer, monitored by control software (XCalibur, Thermo). Raw data was then converted into mzXML format through ProteoWizard software. A self-developed R program package (with XCMS kernel) was used for peak recognition, peak extraction, peak alignment, and integration processing. Subsequently, the data was matched with the secondary mass spectrometry database for metabolites annotation with the Cutoff value for algorithm scoring setting to 0.3.

Following this, the multivariate statistical analysis, hierarchical clustering (HCA), and metabolite correlation analysis on the metabolite data were performed on the metabolite data. The biological significance of the metabolites was elucidated through functional pathways analysis. The multi-omics analysis integrated transcriptomics and metabolomics data using the Joint Pathway Analysis (JPA) module from MetaboAnalyst 5.0, facilitating functional enrichment analysis.

**EdU assay**

To assess cell proliferation, the EdU assay was performed using the Cell-Light EdU Apollo488 *in vitro* imaging kit (Beyotime Biotechnology, Shanghai, China) according to the manufacturer’s instructions. In brief, HRMECs were incubated with EdU at 37℃ for 2 h. Then, they were fixed with 4% PFA and permeated with 0.1% Triton X-100. Subsequently, these cells were stained following the instructions. DAPI was employed to stain cell nuclei for 5 min and the number of EdU-positive cells was observed under a fluorescent microscope at 40× magnification. Quantification of EdU-positive cells was performed using Image J.

**Tube formation assay**

To assess the angiogenic potential of HRMECs, the tube formation assay was conducted. Briefly, 40 μl of growth-factor-reduced Matrigel (BD Biosciences, USA) was pipetted into a pre-cooled 24-well plate and incubated at 37℃ for 25 min for solidification. Following this, cells collected 24 h after transfection were seeded onto the Matrigel and incubated in medium with 10% FBS at 37 ℃ for 4-6 h. Images capturing tube formation were acquired using a light microscope (Olympus, Japan). The Angiogenesis Analyzer of Image J was utilized for quantifying.

**Transwell assay**

The migrating ability of HRMECs was assessed using a transwell assay. Transwell inserts (8-μm pore size, Corning) were employed for migration assay in 24-well plates. The treated cells suspended in 200 μl of serum-free medium were seeded into the upper chamber of each insert. The fresh culture medium with 10% FBS was added to the lower chamber. Following an 16-hour incubation at 37℃, the migrated cells from the upper chambers were fixed with 4% PFA and stained with 0.2% crystal violet. Then, the images were taken using an inverted light microscope (Olympus, Japan) and the number of migrated cells was quantified.

**Wound healing assay**

HRMECs were cultured in 6-well plates until reaching optimal confluency. Following the required treatment, the wound was created in each well using a 10-μl micropipette tip. To eliminate the impact of cell proliferation on the results, serum-free medium was introduced after washing each well with PBS. The cells were then incubated at 37℃ for another 24 h. Changes in open areas at different time points (0 h and 24 h) were captured by an inverted light microscope.

**Reactive oxygen species (ROS) assay**

ROS levels were assessed by a ROS Assay Kit (Beyotime Biotechnology, Shanghai, China) following the manufacturer's instructions. HRMECs were seeded in 24-well plates and transfected with scramble (Scr) siRNA, Odc1 siRNA, or left untreated (Ctrl) for 24 h. Subsequently, GSH was introduced into the Odc1 siRNA group for an additional 24 h. Following this incubation period, the culture medium was removed. DCFH-DA solution was added and incubated at 37 °C for 30 min. The DCFH-DA solution was then removed. Finally, the images were captured using a fluorescence microscope (Olympus, Japan). Image J software was employed for the detection and quantification of fluorescence intensity.

**Statistical analysis**

All data were expressed as mean ± SEM. Each n value corresponded to a single animal for *in vivo* data or an independent experiment for *in vitro* data. The results between experimental groups were analyzed by GraphPad Prism software (version 8.2.1). Two-tailed Student's *t* test or one-way analysis of variance (ANOVA) (if appropriate) was applied to determine statistical significance. *P* value less than 0.05 was considered as statistical significance. To ensure unbiased data analysis in this study, the animals were randomly assigned to experimental groups, minimizing the risk of systematic bias. Blinding was implemented at all stages of this study, including experimental design, animal grouping, experimental processing, and data analysis. In addition, 3 independent analysts conducted data analyses to enhance the robustness of this study. To enhance the reliability, all experiments were repeated at least 4 times. The number of replicates for each experiment was provided in the figure legends, providing clarity on the robustness and consistency of the data.
